# The communities of terrestrial macrofungi in different forest types in vicinities of Khanty-Mansiysk (middle taiga zone of West Siberia)

**DOI:** 10.3897/BDJ.5.e20732

**Published:** 2017-12-29

**Authors:** Nina Filippova, Tatiana Bulyonkova

**Affiliations:** 1 Yugra State University, 628012, Chekhova street, 16, Khanty-Mansiysk, Russia; 2 A.P. Ershov Institute of Informatics Systems Russian Academy of Sciences, 630090, prospekt Lavrentyeva, 6, Novosibirsk, Russia

**Keywords:** biodiversity, macrofungi, funga, boreal forest, West Siberia

## Abstract

**Background:**

The diversity of macrofungi in the vicinities of Khanty-Mansiysk (Yugra, Russia) was surveyed using a method of permanent sampling plots. Ten plots, each consisting of a number of micro-plots, were established in several different communities ranging from old-growth mixed taiga forest to its derivatives in cutting succession and bogged areas. For more complete registration of the mycota, plots were supplemented with random walking routes directly nearby. Survey results were subjected to various quantitative analyses which allowed not only to evaluate the diversity of fungi but also to obtain valuable information on occurrence, abundance and ecology of individual species as well as community structure and its dynamics in the course of ecological succession. The paper reports the results of the first year of observations.

**New information:**

460 species of terrestrial macrofungi revealed in a poorly explored area in middle taiga of West Siberia. The plot-based study revealed differences between communities of terrestrial macrofungi of old coniferous forests, their after-cutting secondary formations and bogged stages. The survey allowed to reveal records of 3 species listed in the Red Data Book of Russia and 9 species listed in the Red Data Book of Yugra.

## Introduction

Terrestrial macrofungi are a crucial component of boreal forest ecosystems playing several significant roles, the most notable being their saprotrophic function and mycorrhizal symbiosis with trees. Basidial macromycetes are the key decomposers of highly recalcitrant coniferous litter; as mycorrhizal partners of trees, they enable the survival of the forest in harsh conditions of the north. The species diversity of terrestrial macrofungi may be several times higher compared to that of plants in coniferous forests and the biomass of mycelium in forest floor is comparable to that of the forest undergrowth (Burova 1986). Terrestrial macrofungi are an important food source of insects and other forest animals; a number of edible and medicinal species are commercially important for man.

Conifers cover vast areas of boreal zone of West Siberia. Low human population density and historical factors have helped to preserve relatively intact massive areas of zonal coniferous forests there. At the same time, the region has always been under-studied mycologically, with most of the research concentrated around large urban scientific centres, notably in Tomsk, Novosibirsk and Yekaterinburg. The region does not have a comprehensive analogue of “Funga Nordica” (Knudsen and Vesterholt 2008); existing checklists are few and only cover fragments of the whole territory with a varying degree of fullness.

While surveying species diversity remains the top priority for our region, it is believed that plot-based studies of fungal communities can yield additional valuable information about the ecology of individual species and about community structure. Thereby, a series of permanent plots for observations of terrestrial macrofungi was laid out in different forest types in the vicinities of Khanty-Mansiysk in the spring of 2015. This paper reports the results of the first year of observations. Although the manpower was limited (only one person was employed on the plots during vegetation season), it is believed that the results can be considered as representative for the surrounding area.

## Materials and methods

### Site description

The studied area is located in the middle taiga zone of West Siberia. The climate is continental subarctic according to the Köppen climate classification. The average annual temperature is –1.3 °C, the mean temperature of the coldest month (January) –19.8 °C, the warmest month is July with its average of 18 °C (Bulatov 2007). Vegetation of the middle taiga zone of West Siberia is characterised by dark coniferous and pine forests and their secondary formations. The forests are made up of spruce (*Picea obovata*), Siberian stone pine (*Pinus sibirica*) and fir (*Abies sibirica*) tree canopy and the undergrowth of various small herbs, ericoid shrubs and feather mosses. The dark coniferous forests are replaced by their secondary communities with *Pinus sylvestris*, *Betula* spp. and *Populus tremula*. The middle taiga of West Siberia is a highly bogged region, with peatlands covering up to 50% of its area. The transition between forests and bogs is represented by pine-birch and pine-dwarfshrub forests (Il'ina et al. 1985).

**Figure 1.**
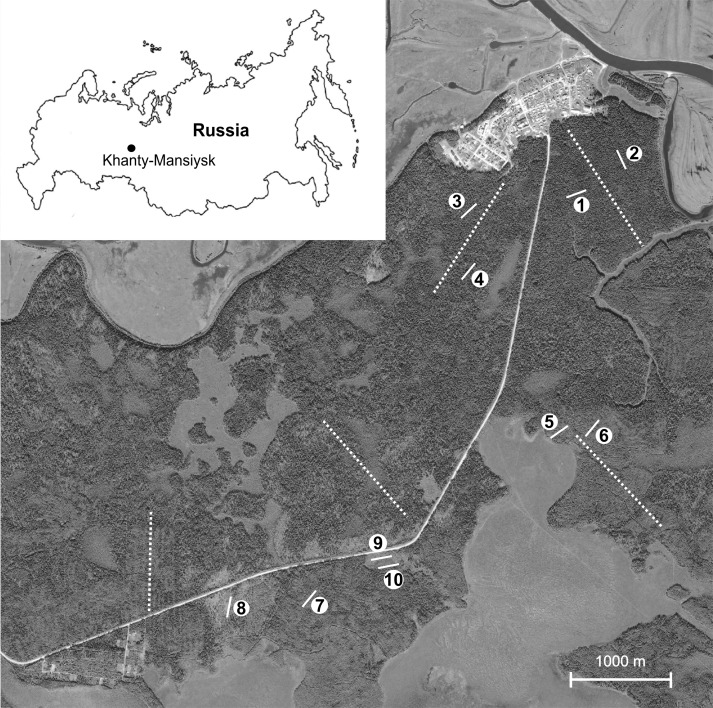
Position of 10 plots (white bars) and walking routes (dotted white lines) on a Landsat satellite image from July 2014 for terrestrial macromycetes observations near Khanty-Mansiysk (Shapsha village showed in upper right corner).

For the purpose of permanent monitoring, an area located 20 km from the Khanty-Mansiysk town was chosen which is at the same time situated within the borders of the natural park «Samarovskiy Chugas» and is relatively intact (Fig. 1). A number of clear-cuts were undertaken here from 5 to 30 years ago which diversified old coniferous forests by their secondary communities. The detailed description of the studied plots is as follows:

Plots 1-4 represent old coniferous forests with dominance of *P. sibirica* with some *P. obovata* and a smaller proportion of *A. sibirica*. Plots 5-7 are located in an old cutting site and represent secondary aspen forests. *P. tremula* dominates in the community with an admixture of *B. pendula* and regrowth of coniferous trees. Plot 8 represents a fresh cutting site where clear-cut was about 5 years ago. The tree layer is absent except for isolated surviving trees (*P. sibirica*, *A. sibirica, P. obovata*) and the regrowth made by *P. tremula*. Plot 9 is located in a small in size bogged locality with waterlogged soil (peat) and residual falling trees. Presumably a fire could have had some impact in the past. Plot 10 is located in proximity to plot 9 on a relatively drier position occupied by wet birch forest. The description of vegetation and geo-reference position of the plots are summarised in Table 1. Photographs of vegetation of studied plots are available under the link: https://www.flickr.com/photos/fungariumysu/albums/72157672157314073.

### Sampling design

Ten permanent plots were established in spring 2015 distributed over an area of about 10 square kilometres Fig. 1. The locations were chosen to assess major homogenous contours of different forest types, e.g. old-growth coniferous forests and their after-cut secondary forests 5 and 20-30 years after cutting, plus a wet birch forest site and a bogged site. Two plots were established in each forest type when the contour was extensive and uniform (plots 1-6). For smaller and relatively mosaic vegetation contours,a single plot for each contour was only applicable (plots 7-10).

Each plot consisted of 20 circular 5 m^2^ micro-plots 5 m apart aligned in a 200 m long line. The observation area of a plot thus equals 100 m^2^ and the total area of plot-observation during the survey was 1000 m^2^. Centres of each micro-plot were marked by plastic poles. A rope was used to draw the outlines of a plot during its examination. The total number of fruiting bodies of each species was counted on each micro-plot. Counted fruitbodies were removed in order to avoid repeated count in the following visits. A few species, where the counting would be impossible due to their high abundance, were counted by estimated number. Some species with densely clustered growth were counted by number of clusters (e.g. *Collybia cirrhata*). In addition to plots observation, walking routes designed to find species not registered in plots (rare species or species with special requirements for environmental conditions) were used. For this, the authors walked along a straight line (using GPS) and collected only new species. No quantitative count was done at this stage. The length of the routes ranged between 500 m to 2 km depending on the abundance of fruiting or weather conditions.

The plots were visited from the end of May (soon after snow melt) until the middle of September (when fungal fruiting was suppressed by the first frosts). The time interval between subsequent visits of each plot ranged between 14-23 days, resulting in total of 5 visits per plot and 5 visits per random route during the season (one visit per month).

The temperature regime of the plots was measured by temperature loggers located in 5 plots of different vegetation types at 5 cm above soil level (Thermochron loggers DS1921G-F5).

### Processing of specimens, identification and analysis of trophic groups

The collection and processing of specimens was done as described in Lodge et al. (2004). Fresh fruiting bodies were wrapped in aluminium foil and carried to the laboratory to be processed on the day of collection. The processing of specimens included: 1) photographing on a photo-studio table, 2) description of vital characters, 3) preliminary microscopy and determination, 4) filling the data in the database, 5) labelling, and 6) drying at 50°C to store in the Fungarium of Yugra State University (collection acronym YSU-F). By the end of the study, the collection amounted to nearly 1500 dried specimens.

The detailed identification was done during the winter following the collection season. Dry specimens were rehydrated in tap water or KOH (10%); dyes and other chemicals (Congo Red, Melzer reagent, ammonia) were applied when necessary. A Zeiss Axiostar microscope with Achromat 5/0.12, 10/0.25, 40/0.65 (dry) and 100/1.25 (oil immersion) objectives was used for microscopical examination. Мicro-photographs were taken with an AxioCam ERc5s digital camera.

Most of the finds were identified using Funga Nordica keys (Knudsen and Vesterholt 2008) and some additional monographs on particular taxa were used when necessary. Fungal authorities are mentioned according to Index Fungorum (last access 12.2016) and Funga Nordica and the classification of the fungal taxa at various taxonomic ranks follows Index Fungorum.

The trophic groups of species were determined from literature (Kovalenko 1980, Burova 1986, Knudsen and Vesterholt 2008). This classification is not strictly unequivocal as some species could be parasitic in the earlier stage of their life cycle and saprotrophic later. Some species could be both saprotrophic and mycorrhizal (Knudsen and Vesterholt 2008). But the number of such cases is not high and usually, these species were referred to a specific group based on an arbitrary decision.

### Statistical analyses

Statistical analyses were performed using EstimateS (Colwell et al. 2012) and open-source R software (R Core Team 2015). Species accumulation and extrapolation curves and their unconditional confidence intervals were computed analytically, using the formulae built in EstimateS software. Clustering and Principal Component Analysis were made in different packages available in the R software, following Borcard et al. (2011). The dissimilarity matrices were built by the chord distance method (“decostand” and “vegdist” functions of “vegan” package (Oksanen et al. 2013). The best method for the cluster analysis was chosen on the basis of a cophenetic correlation computed between original dissimilarity matrices and the cophenetic distances (by means of the function “cophenetic” of package “stats” (R Core Team and contributors worldwide 2017). The method with the highest cophenetic correlation was employed in the analysis (Unweighted Pair-Group Method using arithmetic Averages) (“hclust” function of “stats”). The optimal number of clusters in the dendrogram was estimated by plotting fusion level values, a function “rect.hclust” of “stats” package was used to draw dotted rectangles around clusters (functions in Borcard et al. 2011). Principal Component Analysis was done on a Hellinger pre-transformed abundance data (“decostand” and “rda” functions of “vegan”) using “cleanplot.pca” function of “vegan” package. Rare taxa (singletons and doubletons) were removed prior to the analysis to simplify the results. The Shannon diversity was calculated in R using natural logarithm (“diversity” function of “vegan”).

### Accepted definitions

*Terrestrial macrofungi* – the macrofungi which confine to terrestrial habitat as opposed to wood-inhabiting species representing another prominent community in boreal forests. However, these groups partially overlap and species were recorded growing on mossy old trunks or buried wood within the plots. This study included the following groups in the analysis: Discomycetes, agaricoid members of Agaricales and Boletales and club-like members of Aphyllophorales (following defenitions in Kirk et al. 2008) and some other minority groups.

*Sporocarp density* – number of sporocarps of macrofungi produced in a certain area. The accumulated sporocarp density, calculated as sum of densities of all visits, was used, as well as sporocarps density per visit when discussing phenology.

*Species abundance* – number of micro-plots where species was registered within a plot. The sum values were calculated for each plot based on all visits. This criterion was selected for statistical analyses to align the species that fruit in density with those with scattered fruiting, thus each micro-plot corresponded to a genet of a particular species.

*Species occurrence* – number of plots where the species was registered (maximum – 10 plots).

*Species richness* – number of species registered in a particular plot.

## Data resources

The collection database was imported to Specify 6 (offline) and Specify 7 (available at https://fungariumysu.org/fungarium-ysu-database) software. The plot-based observation database (as Sampling Event Dataset) was uploaded to GBIF and could be available from the page (http://www.gbif.org/dataset/7648036d-fdac-4ebe-8861-9a28134adae1) (Filippova 2017).

## Checklists

### The total species list of terrestrial macrofungi in different forest types in vicinities of Khanty-Mansiysk (including species encountered in plots and during walking routes)

#### Agaricus semotus

Fr.

#### Agrocybe elatella

(P. Karst.) Vesterh.

#### Agrocybe firma

(Peck) Singer

#### Alloclavaria purpurea

(Fr.) Dentinger & D.J. McLaughlin

#### Amanita battarae

(Boud.) Bon

#### Amanita crocea

(Quél.) Singer

#### Amanita fulva

Fr.

#### Amanita porphyria

Alb. & Schwein.

#### Amanita regalis

(Fr.) Michael

#### Amanita vaginatavar.alba

(Sacc.) Romagn.

#### Ampulloclitocybe clavipes

(Pers.) Redhead, Lutzoni, Moncalvo & Vilgalys

#### Armillaria lutea

Gillet

#### Arrhenia acerosa

(Fr.: Fr.) Kühner

#### Arrhenia discorosea

(Pilát) Zvyagina, Alexandrova & Bulyonkova

#### Arrhenia epichysium

(Pers.) Redhead, Lutzoni, Moncalvo & Vilgalys

#### Arrhenia onisca

(Fr.) Redhead, Lutzoni, Moncalvo & Vilgalys

#### Artomyces pyxidatus

(Pers.) Jülich

#### Asterophora lycoperdoides

(Bull.) Ditmar

#### Auriscalpium vulgare

Gray

#### Baeospora myriadophylla

(Peck) Singer

#### Bolbitius titubans

(Bull.: Fr.) Fr.

#### Boletus edulis

Bull.

#### Boletus subtomentosus

L.: Fr

#### Calocera cornea

(Batsch) Fr.

#### Calocera viscosa

(Pers.) Fr.

#### Calocybe gambosa

(Fr.) Singer

#### Cantharellus cibarius

Fr.

#### Chalciporus piperatus

(Bull.) Bataille

#### Chroogomphus rutilus

(Schaeff.: Fr.) O.K. Mill.

#### Ciboria betulicola

J.W. Groves & M.E. Elliott

#### Ciboria caucus

(Rebent.) Fuckel

#### Clavaria flavipes

Pers.

#### Clavariadelphus truncatus

Donk

#### Clavulina coralloides

(L.) J. Schröt.

#### Clavulinopsis laeticolor

(Berk. & M.A. Curtis) R.H. Petersen

#### Clavulinopsis luteoalba

(Rea) Corner

#### Clavulinopsis umbrinella

(Sacc.) Corne

#### Clitocybe agrestis

Harmaja

#### Clitocybe albofragrans

(Harmaja) Kuyper

#### Clitocybe amarescens

Harmaja

#### Clitocybe candicans

(Pers.) P. Kumm.

#### Clitocybe diatreta

(Fr.) P. Kumm.

#### Clitocybe diosma

Einhell.

#### Clitocybe foetens

Melot

#### Clitocybe globispora

Harmaja

#### Clitocybe metachroa

(Fr.) P. Kumm.

#### Clitocybe nebularis

(Batsch) P. Kumm.

#### Clitocybe odora

(Bull.) P. Kumm.

#### Clitocybe regularis

Peck

#### Clitocybe subspadicea

(J.E. Lange) Bon & Chevassut

#### Clitocybe vermicularis

(Fr.) Quél.

#### Clitocybe vibecina

(Fr.) Quél.

#### Clitopilus prunulus

(Scop.) P. Kumm.

#### Collybia cirrhata

(Schumach.) Quél.

#### Collybia cookei

(Bres.) J.D. Arnold

#### Collybia tuberosa

(Bull.) P. Kumm.

#### Coltricia perennis

(L.) Murrill

#### Conocybe apala

(Fr.: Fr.) Arnolds

#### Conocybe aurea

(Jul. Schäff.) Hongo

#### Conocybe filipes

(G.F. Atk.) Kühner

#### Conocybe merdaria

Arnolds & Hauskn.

#### Conocybe mesospora

Kühner & Watling

#### Conocybe semiglobata

Kuhner & Watling

#### Cordyceps militaris

(L.) Fr.

#### Cortinarius acutus

(Pers.) Fr

#### Cortinarius agathosmus

Brandrud, H. Lindstr. & Melot

#### Cortinarius alborufescens

Imler

#### Cortinarius albovariegatus

(Velen.) Melot

#### Cortinarius alboviolaceus

(Pers.) Fr.

#### Cortinarius anomalus

(Fr.) Fr.

#### Cortinarius argutus

Fr.

#### Cortinarius armeniacus

(Schaeff.) Fr.

#### Cortinarius armillatus

(Fr.) Fr.

#### Cortinarius aurantiomarginatus

M.M. Moser

#### Cortinarius balaustinus

Fr.

#### Cortinarius bataillei

(J. Favre ex M.M. Moser) Hil.

#### Cortinarius biformis

Fr.

#### Cortinarius bivelus

(Fr.) Fr.

#### Cortinarius bolaris

(Pers.) Fr

#### Cortinarius brunneus

(Pers.) Fr.

#### Cortinarius camphoratus

(Fr.) Fr.

#### Cortinarius carbunculus

H. Lindstr. & H. Markl.

#### Cortinarius casimiri

(Velen.) Huijsman

#### Cortinarius cicindela

Kytöv., Niskanen & Liimat.

#### Cortinarius cinnamomeus

(L.) Fr.

#### Cortinarius collinitus

(Pers.) Fr.

#### Cortinarius comptulus

M.M. Moser

#### Cortinarius craticius

Fr.

#### Cortinarius croceus

(Schaeff.) Gray

#### Cortinarius decipiens

(Pers.) Fr.

#### Cortinarius delibutus

Fr.

#### Cortinarius depressus

Fr.

#### Cortinarius diasemospermus

Lamoure

#### Cortinarius disjungendus

P. Karst.

#### Cortinarius dolabratus

Fr.

#### Cortinarius duracinus

Fr.

#### Cortinarius evernius

(Fr.) Fr.

#### Cortinarius flexipes

(Pers.) Fr.

#### Cortinarius fusisporus

Kühner

#### Cortinarius gentilis

(Fr.) Fr.

#### Cortinarius glandicolor

(Fr.) Fr.

#### Cortinarius illuminus

Fr.

#### Cortinarius laniger

Fr.

#### Cortinarius lepidopus

Cooke

#### Cortinarius lux-nymphae

Melot

#### Cortinarius melleopallens

(Fr.) Britzelm.

#### Cortinarius multiformis

Fr.

#### Cortinarius obtusus

(Fr.) Fr.

#### Cortinarius ochrophyllus

Fr.

#### Cortinarius paragaudis

Fr.

#### Cortinarius parvannulatus

Kühner

#### Cortinarius pholideus

(Lilj.) Fr.

#### Cortinarius phrygianus

(Fr.) Fr.

#### Cortinarius pilatii

Svrček

#### Cortinarius porphyropus

(Alb. & Schwein.) Fr.

#### Cortinarius praestigiosus

(Fr.) M.M. Moser

#### Cortinarius raphanoides

(Pers.) Fr.

#### Cortinarius sanguineus

(Wulfen) Fr.

#### Cortinarius saturninus

(Fr.: Fr.) Fr.

#### Cortinarius scaurus

(Peck) Brandrud

#### Cortinarius semisanguineus

(Fr.) Gillet

#### Cortinarius septentrionalis

Bendiksen, K. Bendiksen & Brandrud

#### Cortinarius spilomeus

(Fr.) Fr.

#### Cortinarius talus

Fr.

#### Cortinarius tortuosus

(Fr.) Fr.

#### Cortinarius traganus

(Fr.: Fr.) Fr.

#### Cortinarius trivialis

J.E. Lange

#### Cortinarius tubarius

Ammirati & A.H. Sm.

#### Cortinarius umbrinolens

P.D. Orton

#### Cortinarius venustus

P. Karst.

#### Cortinarius vernus

H. Lindstr. & Melo

#### Cortinarius vibratilis

(Fr.) Fr.

#### Cortinarius violaceus

(L.) Gray

#### Crepidotus cesatii

(Rabenh.) Sacc.

#### Crepidotus epibryus

(Fr.) Quél.

#### Crepidotus mollis

(Schaeff.) Staude

#### Cudonia circinans

(Pers.) Fr.

#### Cuphophyllus virgineus

(Wulfen) Kovalenko

#### Cystoderma amianthinum

(Scop.) Fayod

#### Cystoderma carchariasvar.fallax

(Pers.: Fr.) Fayod

#### Cystoderma jasonis

(Cooke & Massee) Harmaja

#### Cystodermella adnatifolia

(Peck) Harmaja

#### Cystodermella cinnabarina

(Alb. & Schwein.) Harmaja

#### Cystodermella granulosavar.granulosa

(Batsch) Harmaja

#### Cystolepiota seminuda

(Lasch) Bon

#### Dendrocollybia racemosa

(Pers.) R.H. Petersen & Redhead

#### Elaphomyces asperulus

Vittad.

#### Entoloma allospermum

Noordel.

#### Entoloma cetratum

(Fr.) M.M. Moser

#### Entoloma conferendum

(Britzelm.) Noordel.

#### Entoloma depluens

(Batsch) Hesler

#### Entoloma lampropus

(Fr.) Hesler

#### Entoloma lanuginosipes

Noordel.

#### Entoloma myrmecophilum

(Romagn.) M.M. Moser

#### Entoloma nitens

(Velen.) Noordel.

#### Entoloma rhodopoliumvar.pseudopolitum

(Fr.) P. Kumm.

#### Entoloma rusticoides

(Gillet) Noordel.

#### Entoloma sericatum

(Britzelm.) Sacc

#### Entoloma sericellum

(Fr.) P. Kumm.

#### Entoloma sericeum

Quél.

#### Entoloma solstitiale

(Fr.) Noordel.

#### Flammulaster rhombosporus

(G.F. Atk.) Watling

#### Flammulaster subincarnatus

(Joss. & Kühner) Watling

#### Flammulina elastica

(Lasch) Redhead & Petersen

#### Galerina allospora

A.H. Sm. & Singer

#### Galerina atkinsoniana

A.H. Sm.

#### Galerina camerina

(Fr.) Kühner

#### Galerina cephalotricha

Kühner

#### Galerina cerina

A.H. Sm. & Singer

#### Galerina hypnorum

(Schrank)

#### Galerina marginata

(Batsch)

#### Galerina mniophila

(Lasch: Fr.)

#### Galerina paludosa

(Fr.)

#### Galerina pumila

(Pers.) M. Lange

#### Galerina salicicola

P.D. Orton

#### Galerina vittiformis

(Fr.) Singer

#### Gamundia hygrocyboides

(Lonati) Bon

#### Gamundia striatula

(Kühner) Raithelh.

#### Gomphidius flavipes

Peck

#### Gomphus clavatus

(Pers.) Gray

#### Grifola frondosa

(Dicks.) Gray

#### Gymnopilus penetrans

(Fr.) Murrill

#### Gymnopilus sapineus

(Fr.) Murrill

#### Gymnopus androsaceus

(L.) Della Maggiora & Trassinelli

#### Gymnopus confluens

(Pers.: Fr.) Antonn, Halling & Noordel.

#### Gymnopus dryophilus

(Bull.) Murrill

#### Gymnopus inodorus

(Pat.) Antonín & Noordel.

#### Gymnopus ocior

(Pers.) Antonín & Noordel.

#### Gymnopus peronatus

(Bolton) Gray

#### Gymnopus putillus

(Fr.: Fr.) Antonn, Halling & Noordel.

#### Gyromitra esculenta

(Pers.) Fr.

#### Gyromitra gigas

(Krombh.) Cooke

#### Gyromitra infula

(Schaeff.) Quél.

#### Hebeloma fragilipes

Romagn.

#### Hebeloma hiemale

Bres.

#### Hebeloma incarnatulum

A.H. Sm.

#### Hebeloma radicosum

(Bull.) Ricken

#### Hebeloma sordescens

Vesterh.

#### Hebeloma velutipes

Bruchet

#### Helvella cupuliformis

Dissing & Nannf.

#### Helvella lacunosa

Afzel.

#### Helvella macropus

(Pers.) P. Karst.

#### Hemimycena delectabilis

(Peck) Singer

#### Hemimycena mairei

(J.-E. Gilbert) Singer

#### Hemimycena pseudocrispula

(Kühner) Singer

#### Hemimycena sordida

Noordel. & Antonín

#### Hemimycena subtilis

(Velen.) Antonín

#### Hericium cirrhatum

(Pers.) Nikol.

#### Hericium coralloides

(Scop.) Pers.

#### Hohenbuehelia nigra

(Schwein.) Singer

#### Hohenbuehelia petalodes

(Bull.: Fr.) Schulzer

#### Humaria hemisphaerica

(F.H. Wigg.) Fuckel

#### Hydnum repandum

L.

#### Hydnum rufescens

Pers.

#### Hygrocybe cantharellus

(Schwein.: Fr.) Murrill

#### Hygrocybe conicavar.conica

(Schaeff.) P. Kumm.

#### Hygrocybe constrictospora

Arnolds

#### Hygrocybe glutinipesvar.glutinipes

(J.E. Lange) R. Haller Aar.

#### Hygrocybe laeta

(Pers.: Fr.) P. Kumm.

#### Hygrocybe reidii

Kühner

#### Hygrophorus erubescens

(Fr.) Fr.

#### Hygrophorus olivaceoalbus

(Fr.) Fr.

#### Hygrophorus piceae

Kühner

#### Hymenochaete tabacina

(Sowerby) Lév.

#### Hypholoma capnoides

(Fr.) P. Kumm.

#### Hypholoma ericaeoides

P.D. Orton

#### Hypholoma fasciculare

(Huds.) P. Kumm.

#### Hypholoma marginatum

(Pers.: Fr.) J. Schröt.

#### Hypholoma polytrichi

(Fr.) Ricken

#### Hypocrea gelatinosa

(Tode) Fr.

#### Hypomyces luteovirens

(Fr.) Tul. & C. Tul.

#### Hypsizygus ulmarius

(Bull.) Redhead

#### Infundibulicybe gibba

(Pers.) Harmaja

#### Infundibulicybe squamulosa

(Pers.) Harmaja

#### Inocybe albovelutipes

Stangl

#### Inocybe amethystina

Kuyper

#### Inocybe cookei

Bres.

#### Inocybe geophylla

(Bull.) P. Kumm.

#### Inocybe griseolilacina

J.E. Lange

#### Inocybe jacobi

Kühner

#### Inocybe lacera

(Fr.) P. Kumm.

#### Inocybe lanuginosa

(Bull.) P. Kumm.

#### Inocybe maculata

Boud.

#### Inocybe mixtilis

(Britzelm.) Sacc.

#### Inocybe perlata

(Cooke) Sacc.

#### Inocybe phaeodisca

Kühner

#### Inocybe putilla

Bres.

#### Inocybe subcarpta

Kühner & Boursier

#### Inocybe subnudipes

Kühner

#### Inocybe urceolicystis

Stangl & Vauras

#### Kuehneromyces lignicola

(Peck) Redhead

#### Kuehneromyces mutabilis

(Schaeff.) Singer & A.H. Sm.

#### Laccaria bicolor

(Maire) P.D. Orton

#### Laccaria proxima

(Boud.) Pat.

#### Lactarius aurantiacus

(Pers.) Gray

#### Lactarius auriolla

Kytöv.

#### Lactarius deterrimus

Gröger

#### Lactarius flexuosus

(Pers.) Gray

#### Lactarius glyciosmus

(Fr.: Fr.) Fr

#### Lactarius helvus

(Fr.: Fr.) Fr.

#### Lactarius hysginoides

Korhonen & T. Ulvinen

#### Lactarius leonis

Kytöv.

#### Lactarius mammosus

Fr.

#### Lactarius musteus

Fr.

#### Lactarius plumbeus

(Bull.) Gray

#### Lactarius pubescens

Fr.

#### Lactarius repraesentaneus

Britzelm.

#### Lactarius rufus

(Scop.) Fr.

#### Lactarius torminosus

(Schaeff.) Gray

#### Lactarius trivialis

(Fr.) Fr.

#### Lactarius utilis

(Weinm.) Fr.

#### Lactarius uvidus

(Fr.) Fr.

#### Lactarius vietus

(Fr.) Fr.

#### Laetiporus sulphureus

(Bull.) Murrill

#### Leccinum albostipitatum

den Bakker & Noordel.

#### Leccinum aurantiacum

(Bull.) Gray

#### Leccinum scabrum

(Bull.) Gray

#### Leccinum versipelle

(Fr.) Snell

#### Lentinellus micheneri

(Berk. & M.A. Curtis) Pegler

#### Lentinus vulpinus

(Sowerby: Fr.) Kühner & Maire

#### Leotia lubrica

(Scop.) Pers.

#### Lepiota clypeolaria

(Bull.) P. Kumm.

#### Lepiota cristata

(Bolton) P. Kumm.

#### Lepiota felina

(Pers.) P. Karst.

#### Lepista sordida

(Schumach.: Fr.) Singer

#### Lichenomphalia umbellifera

(L.) Redhead, Lutzoni, Moncalvo & Vilgalys

#### Lycoperdon molle

Pers.

#### Lycoperdon nigrescens

Pers.

#### Lycoperdon perlatum

Pers.

#### Lycoperdon pyriforme

Schaeff.

#### Lycoperdon umbrinum

Pers.

#### Macrotyphula fistulosa

(Holmsk.) R.H. Petersen

#### Macrotyphula juncea

(Alb. & Schwein.) Berthier

#### Marasmius epiphyllus

(Pers.) Fr.

#### Marasmius rotula

(Scop.) Fr.

#### Megacollybia platyphylla

(Pers.) Kotl. & Pouzar

#### Melanoleuca melaleuca

(Pers.) Murrill

#### Melanoleuca polioleuca

(Fr.: Fr.) Kühner & Maire

#### Melanoleuca strictipes

(P. Karst.) Jul. Schff.

#### Mycena acicula

(Schaeff.) P. Kumm.

#### Mycena aciculata

(A.H. Sm.) Desjardin & E.

#### Mycena adonis

(Bull.: Fr.) Gray

#### Mycena algeriensis

Maire

#### Mycena amicta

(Fr.) Quél.

#### Mycena capillaripes

Peck

#### Mycena citrinomarginata

Gillet

#### Mycena clavicularis

(Fr.) Gillet

#### Mycena delectabilis

(Peck) Singer

#### Mycena epipterygia

(Scop.) Gray

#### Mycena erubescens

Höhn.

#### Mycena fibula

(Bull.) Kühner

#### Mycena fragillima

A.H. Sm.

#### Mycena galopus

(Pers.) P. Kumm.

#### Mycena haematopus

(Pers.) P. Kumm

#### Mycena hiemalis

(Osbeck) Quél.

#### Mycena laevigata

(Lasch) Gillet

#### Mycena leptocephala

(Pers.) Gillet

#### Mycena metata

(Secr. ex Fr.) P. Kumm.

#### Mycena mirata

(Peck) Sacc.

#### Mycena niveipes

(Murrill) Murrill

#### Mycena olida

Bres.

#### Mycena olivaceomarginata

(Massee) Massee

#### Mycena pelianthina

(Fr.) Quél.

#### Mycena pseudopicta

(J.E. Lange) Kühner

#### Mycena pura

(Pers.) P. Kumm

#### Mycena silvae-nigrae

Maas Geest. & Schwöbel

#### Mycena stipata

Geest. & Schwöbel

#### Mycena stylobates

(Pers.: Fr) P. Kumm.

#### Mycena subcana

A.H. Sm.

#### Mycena tristis

Maas Geest.

#### Mycena viridimarginata

P. Karst.

#### Mycena vitilis

(Fr.) Quél.

#### Mycena vulgaris

(Pers.: Fr.) P. Kumm.

#### Mycenella lasiosperma

(Bres.) Locq.

#### Mycetinis querceus

(Britzelm.) Antonín & Noordel.

#### Myxomphalia maura

(Fr.) Hora

#### Neolentinus cyathiformis

(Schaeff.) Della Maggiora & Trassinelli

#### Neolentinus lepideus

(Fr.) Redhead & Ginns

#### Omphaliaster asterosporus

(J.E. Lange) Singer

#### Ophiocordyceps gracilis

(Grev.) Durieu & Mont.

#### Ossicaulis lignatilis

(Pers.) Redhead & Ginns

#### Otidea leporina

(Batsch) Fuckel

#### Otidea platyspora

Nannf.

#### Panus neostrigosus

Drechsler-Santos & Wartchow

#### Paxillus involutus

(Batsch) Fr.

#### Peziza arvernensis

Roze & Boud.

#### Peziza michelii

(Boud.) Dennis

#### Pholiota astragalina

(Fr.) Singer

#### Pholiota flammans

(Batsch) P. Kumm.

#### Pholiota lenta

(Pers.) Singer

#### Pholiota limonella

(Peck) Sacc.

#### Pholiota lubrica

(Pers.) Singer

#### Pholiota spumosa

(Fr.) Singer

#### Pholiota squarrosa

(Vahl) P. Kumm.

#### Pholiota subochracea

(A.H. Sm.) A.H. Sm. & Hesler

#### Pholiotina parvula

(Døssing & Watling) Bon

#### Pleurotus pulmonarius

(Fr.: Fr.) Quél.

#### Pluteus cervinus

(Schaeff.) P. Kumm.

#### Pluteus chrysophaeus

(Schaeff.) Quél.

#### Pluteus cyanopus

Quél.

#### Pluteus exiguus

(Pat.) Sacc.

#### Pluteus leoninus

(Schaeff.) P. Kumm.

#### Pluteus nanus

(Pers.) P. Kumm.

#### Pluteus petasatus

(Fr.) Gillet

#### Pluteus phlebophorus

(Ditmar) P. Kumm.

#### Pluteus plautus

(Weinm.) Gillet

#### Pluteus podospileus

Sacc. & Cub.

#### Pluteus pouzarianus

Singer

#### Pluteus romellii

(Britzelm.) Sacc.

#### Pluteus salicinus

(Pers.) P. Kumm.

#### Pluteus semibulbosus

(Lasch) Quél.

#### Pluteus umbrosus

(Pers.) P. Kumm

#### Polyporus ciliatus

Fr.

#### Polyporus melanopus

(Pers.) Fr.

#### Polyporus squamosus

(Huds.) Fr.

#### Polyporus varius

(Pers.) Fr.

#### Psathyrella caput-medusae

(Fr.) Konrad & Maubl.

#### Psathyrella fatua

(Fr.) Konrad & Maubl.

#### Psathyrella fusca

(Schumach.) A. Pearson

#### Psathyrella larga

(Kauffman) A.H. Sm.

#### Psathyrella pygmaea

(Bull.: Fr.) Singer

#### Psathyrella squamosa

(P. Karst.) A.H. Sm.

#### Pseudoclitocybe cyathiformis

(Bull.) Singer

#### Pseudoomphalina pachyphylla

(Fr.) Knudsen

#### Pseudoplectania melaena

(Fr.) Sacc.

#### Pseudoplectania nigrella

(Pers.) Fuckel

#### Psilocybe inquilinus

(Fr.: Fr.) Bres.

#### Psilocybe montana

(Pers.: Fr.) P. Kumm.

#### Psilocybe phyllogena

(Sacc.) Peck

#### Psilocybe turficola

J. Favre

#### Pterula multifida

(Chevall.) Fr.

#### Ramaria pallida

(Schaeff.) Ricken

#### Ramaria tsugina

(Peck) Marr & D.E. Stuntz

#### Ramariopsis asperulospora

(G.F. Atk.) Corner

#### Ramariopsis crocea

(Pers.) Corner

#### Ramariopsis subtilis

(Pers.) R.H. Petersen

#### Rhodocollybia butyraceavar.asema

(Bull.) Lennox

#### Rhodocollybia fodiens

(Kalchbr.) Antonín & Noordel.

#### Rhodocollybia maculatavar.maculata

(Alb. & Schwein.) Singer

#### Rickenella fibula

(Bull.: Fr.) Raithelh.

#### Rickenella swartzii

(Fr.: Fr.) Kuyper

#### Roridomyces roridus

(Fr.) Rexer

#### Russula acrifolia

Romagn.

#### Russula aeruginea

Lindblad

#### Russula amethystina

Quél.

#### Russula aquosa

Leclair

#### Russula atroglauca

Einhell.

#### Russula badia

Quél.

#### Russula claroflava

Grove

#### Russula consobrina

(Fr.) Fr.

#### Russula decolorans

(Fr.: Fr.) Fr.

#### Russula depallens

(Pers.: Fr.) Fr.

#### Russula emetica

emetica (Schaeff.: Fr.) Pers.

#### Russula foetens

Pers.

#### Russula gracillima

Jul. Schäff.

#### Russula grisescens

(Bon & Gaugué) Marti

#### Russula medullata

Romagn.

#### Russula puellaris

Fr.

#### Russula renidens

Ruots., Sarnari & Vauras

#### Russula rhodopus

Zvára

#### Russula rivulicola

Ruots. & Vauras

#### Russula sapinea

Sarnari

#### Russula sphagnophila

Kauffman

#### Russula torulosa

Bres.

#### Russula versicolor

Jul. Schäff.

#### Russula xermpelina

(Schaeff.) Fr.

#### Sarcosoma globosum

(Schmidel) Casp.

#### Schizophyllum amplum

(Lév.) Nakasone

#### Simocybe sumptuosa

(P.D. Orton) Singer

#### Spathularia rufa

Schmidel

#### Sphaerobolus stellatus

Tode

#### Strobilurus stephanocystis

(Hora) Singer

#### Strobilurus tenacellus

(Pers.: Fr.) Singer

#### Stropharia albonitens

(Fr.) Quél.

#### Stropharia hornemannii

(Fr.) S. Lundell & Nannf.

#### Stropharia pseudocyanea

(Desm.: Fr.) Morgan

#### Suillus acidus

(Peck) Singer

#### Suillus pictus

(Peck) Kuntze

#### Suillus placidus

(Bonord.) Singer

#### Suillus sibiricus

(Singer) Singer

#### Suillus variegatus

(Sw.) Richon & Roze

#### Tephrocybe rancida

(Fr.) Donk

#### Trichoglossum hirsutum

(Pers.) Boud.

#### Tricholoma fulvum

(DC.) Bigeard & H. Guill

#### Tricholoma inamoenum

(Fr.: Fr.) Gillet

#### Tricholoma rapipes

(Krombh.) Heil.-Claus. & Mort.

#### Tricholoma stiparophyllum

(N. Lund) P. Karst.

#### Tricholoma sudum

(Fr.) Quél.

#### Tricholoma virgatum

(Fr.) P. Kumm.

#### Tricholoma viridilutescens

M.M. Moser

#### Tricholomopsis decora

(Fr.: Fr.) Singer

#### Tricholomopsis rutilans

(Schaeff.) Singer

#### Trichophaeopsis bicuspis

(Boud.) Korf & Erb

#### Tubaria confragosa

(Fr.) Harmaja

#### Tubaria conspersa

(Pers.) Fayod

#### Tubaria furfuracea

(Pers.) Gillet

#### Tylopilus felleus

(Bull.) P. Karst.

#### Typhula erythropus

(Pers.) Fr.

#### Xeromphalina campanella

(Batsch) Kühner & Maire

#### Xeromphalina fraxinophila

A.H. Sm.

## Analysis

### Taxonomic, ecological structure and species richness curves

The final list of macrofungi in the vicinities of Khanty-Mansiysk has encompassed 460 species from 6 classes, 14 orders, 55 families and 130 genera. The Agaricales is the richest order comprising 75% of the total species number, followed by Russulales (10%), Boletales (3%), Pezizales (3%), Polyporales (2%) and others in minority.

The total number of species revealed during plots observations reached 313, which represents 2/3 of the total species list revealed during the survey. Another 147 species were not registered inside permanent observation plots but were found during random routes laid out in the vicinities of the plots. The number of species differed substantially between plots: it was the lowest in the bogged site (plot 9) – 21 species/100 m^2^ and the highest in the fresh cutting site (plot 8) – 72 species/100 m^2^. The average number of species between four plots in dark coniferous forests (plots 1-4) was 67 species and the average species number in secondary forests was 56.

The Shannon index, calculated for species abundances in plots, varied between 2.7 and 4.1, where the minimum value corresponded to the bogged site due to low species number and other 9 plots had close values, on average = 3.8.

The total number of sporocarps counted during the field season (=density per season) reached 5309 sporocarps (e.g. 5309 sporocarps per vegetation season/1000 m^2^) Table 2. The sporocarps density changed from the beginning to the end of summer, being low in May and June (163 and 154 sporocarps/month/1000 m^2^), highest in July (2283) and subsided in August and September (1207 and 1502 sporocarps/month/1000 m^2^). The sporocarps density differed between plots: it was highest in fresh cutting site (plot 8) – 1010 sporocarps/100 m^2^/season and lowest in aspen forest (plot 5) and bogged site (plot 9) – 307 and 340 sporocarps/100 m^2^/season respectively. The sporocarps density of seven other plots was approximately 500/100 m^2^/season.

The analysis of sporocarps density per season within micro-plots (5 m^2^) revealed a high standard deviation between different micro-plots of the same plot and absence of statistically significant differences between densities of different plots. An analysis of number of species by micro-plots showed similarly high standard deviation. The average number of species within a micro-plot differed significantly only between bogged site and some other plots. The statistical analysis using micro-plots counts is not fully justified, however, because the size of micro-plots is too small to fit the community.

**Figure 2.**
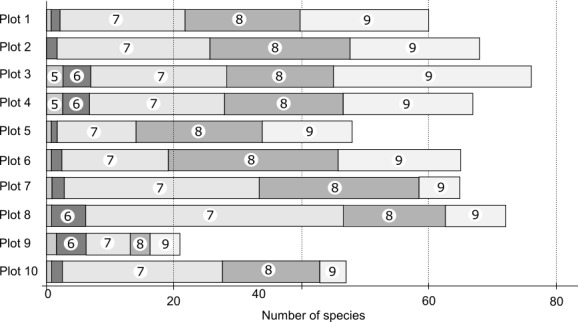
Total number of species by plots and relative proportion of species number by months within each plot (numbers refer to the corresponding months).

The total species number changed over the season, being 9, 24, 161, 140 and 115 from May to September respectively. The species number was equally high in July, August and September on all plots, but there were less species in September in aspen and birch forests and relatively higher number of species in July in fresh cutting sites (Fig. 2).

Regarding the phenology of individual species, 218 species were found with expressed seasonal dynamics. Examples of species fruiting in early summer (May-June) are: *Entoloma sericeum*, *Pseudoplectania nigrella*, *Mycena viridimarginata* and others. Some species were fruiting in mid-summer (July), for example: *Armillaria lutea*, *Galerina vittiformis*, *Mycena epipterygia*; and some in late summer (August-September): *Cortinarius decipiens*, *C. parvannulatus*, *Inocybe urceolicystis*, *Crepidotus epibryus*. Other species could have prolonged fruiting throughout the whole of the vegetation season, or intermediate between mentioned types.

**Figure 3.**
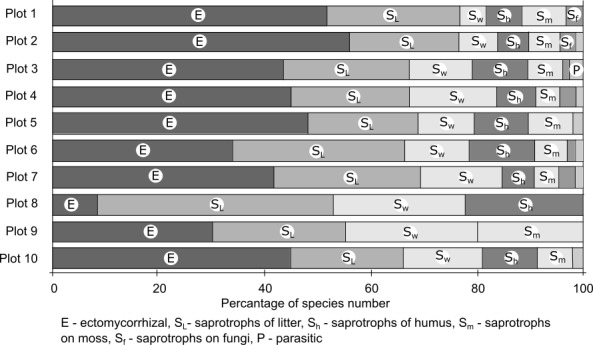
Relative proportion of different trophic groups within each plot.

The analysis of the total species list by trophic groups showed that saprotrophic species prevailed (60%) with the rest being ectomycorrhizal (38%) and parasitic (2%) species (Fig. 3). One hundred and thirty (130) ectomycorrhizal species formed association with 9 tree species (4 coniferous and 5 deciduous trees), thus the average host/symbiont ratio was about 1:18 (excluding two rare species of Salix).

Within the class of saprotrophic species, the prevailing group is saprotrophs of litter layer (45%), followed by lignicolous species (26%), saprotrophs on humus layer (19%), saprotrophs on moss (7%) and on mushrooms (3%). The trophic structure differs between the plots: the percentage of saprotrophic and ectomycorrhizal species was nearly equal in plots 1-5, 7 and 10 (all coniferous forests, one of two plots in aspen forest and birch forest). The number of saprotrophic species was twice as high in one of two plots in birch-aspen forest (plot 6) and three times higher in the bogged site (plot 9). Contrastingly, the percentage of saprotrophic species in the fresh cutting site (plot 8) reached 92% with mycorrhizal species being in minority. The proportion of groups within the saprotrophic class was nearly equal between plots except for the absence of saprotrophs of the humus layer in the bogged site and the absence of saprotrophs on moss in the fresh cutting site.

Within the class of ectomycorrhizal species, the largest group was symbionts of coniferous trees (40%), followed by an equal number of species of deciduous (30%) and broad spectrum symbionts (30%).

**Figure 4.**
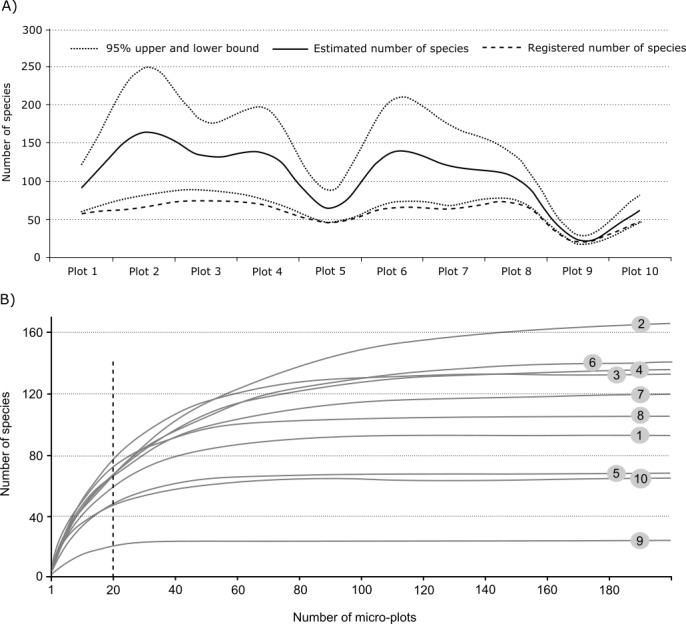
Estimates species richness and species accumulation curves for 10 plots of macromycetes observations in different forest types in vicinities of Khanty-Mansiysk, West Siberia: A) Estimated species richness with 95% upper and lower limits and registered species number, B) Species accumulation curves built on 20 observed micro-plots (dotted line) with extrapolation up to 200 micro-plots (1000 m^2^) (confidence interval not shown in the picture).

The species accumulation curves built for all 10 plots individually with extrapolations to a total of 200 micro-plots allowed comparison of predicted species richness (Fig. 4). Confidence intervals of estimated species richness overlapped for all plots except for plot 9. This means that the estimated species richness does not differ between studied plots except for the bogged site, where species richness was much less than in the others. The revealed species number was within the lower estimated boundary in plots 1, 5, 9 and 10, thus the species richness in these plots is sufficiently revealed. The species richness is expected to grow with increasing area of observations for all other plots. As the four sites in coniferous forests were fairly uniform (plots 1-4), a composite species accumulation curve was built based on 100 micro-plots of these plots collectively with extrapolation to a total of 400 micro-plots. The predicted species richness was 250 (with lower and upper limits being 200 and 300), while registered species richness was 166 species. Thus, the total observation area of 400 m^2^ in coniferous forests is still not enough for a complete survey of species richness. Finally, the estimated species richness of the total area based on cumulative samples of all plots equals 409 (from 368 to 450), while registered species richness during plot observations was 313. However, the total species richness revealed during the projects (through plots observations + random routes) was exactly 460, which is close to the upper limit of predicted species richness.

### Analysis of fungal community structure in different forest types

**Figure 5.**
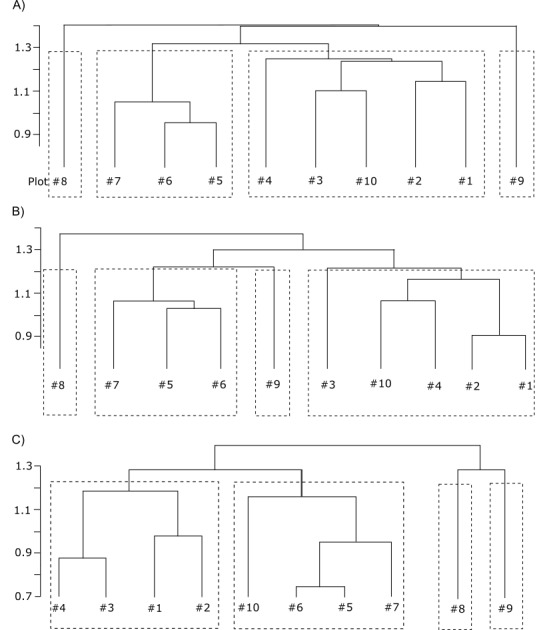
Cluster dendrograms built by average agglomerative clustering (UPGMA) based on chord distance matrix of abundance data of fungi amongst 10 plots of macromycetes observations in different forest types in vicinities of Khanty-Mansiysk, West Siberia: A) all trophic groups altogether, B) separate analysis for saprotrophic species, C) separated analysis for ectomycorrhizal species.

**Figure 6.**
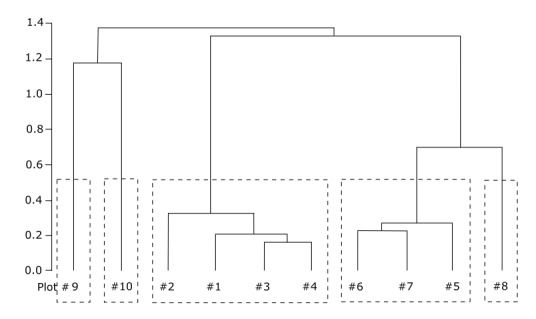
Cluster dendrogram built by average agglomerative clustering (UPGMA) based on chord distance matrix of abundance data of vegetation amongst 10 plots of macromycetes observations in different forest types in vicinities of Khanty-Mansiysk, West Siberia.

The cluster analysis helped to reveal a logical classification of ten studied plots on the basis of composition and abundance of fungal species, although it was difficult to make this classification manually (Fig. 5). The fresh cutting site and the bogged site had the greatest difference with other clusters, where secondary forests (plots 5-7) made a common cluster and old coniferous forests (plots 1-4) made a second cluster. Besides, the predominantly birch forest site (plot 10) has entered the cluster of coniferous plots and was closer to plot 3. The authors decided to compare the results of clustering of fungal communities with vegetation clustering based on botanical descriptions made on the same plots (Fig. 6). The vegetation plots within coniferous cluster (plots 1-4) and within secondary forest (5-7) were merged on a lower height compared to corresponding fungal plots, which indicates a greater homogeneity amongst vegetation than amongst fungal community within the same plots. However, the clusters of higher level were merged on the same height for vegetation as for fungal community. Three plots (8-10) were differently merged in both dendrograms: plots 8 and 9 were separated from the rest of the plots at a high level in the fungal dendrogram, while plot 8 is merged with the deciduous cluster and plots 9 and 10 separated from the rest at a high level in the vegetation dendrogram. In other words, the fungal community differs very markedly during the first stages of after-cut succession compared to vegetation, which stays stable for longer periods (plot 8, five-years ago cutting site vs. plots 5-7, thirty-forty years secondary forests). Regarding plots 9-10, they are located in close proximity and represent successive stages of bogging where former coniferous forest is replaced by wet predominantly birch forest and finally by bogged site. The vegetation on these two plots is relatively similar, while the fungal community of plot 10 is inclined to a coniferous cluster (and former coniferous forests inhabiting this particular site). The fungal community of the bogged site is very different from the rest of the plots, showing bogging as a powerful factor affecting fungal community structure.

Additionally, the cluster analysis of fungal communities was made separately for saprotrophic and ectomycorrhizal species (Fig. 5: B, C). The dendrogram of saprotrophic species was close to the general dendrogram described above except for the position of plot 9 (bogged site) which was united in a common cluster with secondary plots. In the dendrogram of ectomycorrhizal species, the position of plot 10 moved from the coniferous cluster (in general dendrogram) to the secondary cluster.

**Figure 7.**
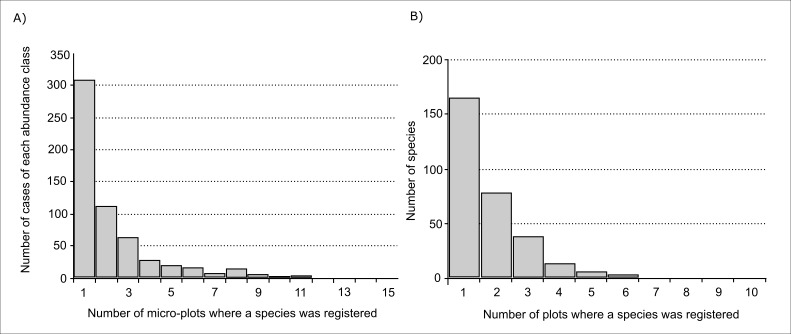
The abundance and occurence distribution: A) histogram of species abundances, B) histogram of species occurrences.

The abundance and occurrence distribution of species are important characteristics of the community. When considering abundance distribution, the number of cases of singletons (registration in only one micro-plot within the plot) was the highest, the number of cases when species occurred in 2-9 micro-plots was two times lower and the number of cases when the species occurred in more than half plots was only 13 (Fig. 7A). For example, species with abundance >10 (more than 10 micro-plots within a single plot) were: *Russula sphagnophila* (15), *Armillaria lutea* (14), *Cortinarius flexipes* (13), *C. tubarius* (13), *C. decipiens* (12) , *Galerina atkinsoniana* (12), *Conocybe semiglobata* (11), *Lactarius vietus* (11). If all plots are considered altogether, the number of singletons (the species found only in one from the total 200 micro-plots) reaches 113 species (or 36% of the total species list) and the number of doubletons is 53 (17%). Thus, the number of rare species (where singletons and doubletons for all plots are included altogether) represents half of the species list revealed during plots observations. Additionally, 147 species registered during additional random surveys in plots vicinities could also be classified as rare (although without quantitative data). The prior list of rarely registered species therefore reaches 313 species, or 68% or the total list.

The occurrence of species characterises its presence in all or only part of the studied plots; about half of the species (53%) were registered in only one plot, 45% were registered in 2-5 plots and only 3% of species were registered in more than half of the plots (Fig. 7). These ubiquitous species were: *Cortinarius decipiens* (occurred in 6 plots), *C. parvannulatus* (6), *C. raphanoides* (7), *Galerina atkinsoniana* (9), *Mycena mirata* (10), *M. viridimarginata* (8), *Psilocybe montana* (6) and *P. phyllogena* (6).

**Figure 8.**
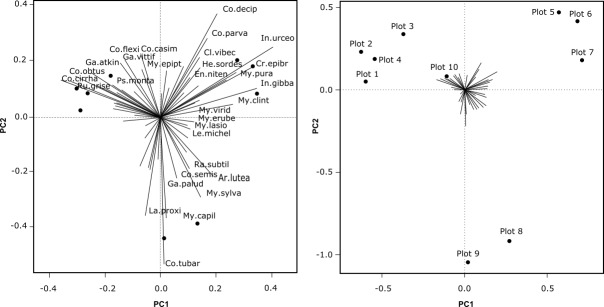
PCA analysis of pre-transformed species abundance matrix for 10 plots of macromycetes observations in different forest types in vicinities of Khanty-Mansiysk, West Siberia. Left: Scaling 1 biplot showing species contribution to axes 1 and 2 (only species with high contribution shown), Right: Scaling 2 biplot showing grouping of plots on the axes 1 and 2 (species were omitted from the biplot for clarity).

Concerning the question of ubiquity of some species, it is interesting to consider restriction of species to a particular community. To answer the question, a Principal Component Analysis was performed on pre-transformed species abundance data (single- and doubletons were previously removed to simplify the process). The grouping of plots on Scaling 2 biplot corresponds to four clusters made by cluster analysis (Fig. 8). The Scaling 1 biplot shows how the species contribute to axes 1 and 2 (species with more contribution have longer vectors). From the table ordered by PCA results, the fresh cutting site (plot 8) was characterised by a large group of species which rarely or do not occur in other plots (*Armillaria lutea*, *Ramariopsis subtilis*, *Sphaerobolus stellatus*, *Psathyrella fatua*, *Psilocybe inquilina*, *Conocibe semiglobata*, *Galerina salicina*, *Pluteus cervinus*, *Conocybe merdaria*, *Galerina marginata*, *Agrocybe elatella*, *Mycena capillaripes*, *Mycena leptocephala*, *Entoloma sericellum* and *Roridomyces rorida*). The bogged forest site had smallest list of strongly characteristic species (*Cortinarius tubarius*, *Galerina cerina*, *Galerina paludosa* and *Lichenomphalia umbellifera*). The secondary aspen forests were characterised by some species (*Inocybe urceolicystis*, *Collybia cookei*, *Crepidotus epibryus*, *Inocybe gibba* and *Mycena pura*). Two species of ubiquitous occurrence (*C. decipiens* and *C. parvannulatus*) could be also considered characteristic of secondary forests due to their high abundance. Finally, the large cluster of five plots (coniferous old forests, plots 1-4 and wet birch forest, plot 10) has a large list of characteristic species (*Collybia cirrhata*, *Cortinarius flexipes*, *Cortinarius obtusus*, *Russula grisea*, *Psilocybe montana*, *Cortinarius collinitus*, *Galerina vittiformis*, *G. atkinsoniana*, *Cortinarius brunneus*, *Inocybe putilla*, *Hebeloma velutipes*, *Entoloma sericatum*, *Mycena epipterygia*, *Ampulloclitocybe clavipes*, *Entoloma lanuginosipes*, *Psilocybe phyllogena* and *Lactarius vietus*). The birch site (plot 10) has its own characteristic species that does not occur in the rest of the plots of the cluster (*Russula sphagneti*, *Clitocybe candicans*, *Mycena pseudopicta*, *Rickinella fibula*, *Entoloma rhodopolium* and *Gymnopus peronatus*).

## Discussion

### Methodology

The methodology of plot-based studies of macromycetes has been developed by many authors (Arnolds 1992, Burova 1986, Kalamees 1968) and the general approach is recommended nowadays by Lodge et al. 2004. Local conditions and constraints require nevertheless developing individual approaches for a particular project. Several questions of sampling and analysis design are the most important and these will be addressed below: plot size, plot replication, frequency of visits and the method forquantitative counting.

The plot size for fungal permanent plots should be larger than for vegetation due to the сoarser mosaic of fungal communities and recommended to be 1000 m^2^ for forests and about 500 m^2^ for grass- and heathlands (Arnolds 1992, Lodge et al. 2004). In thisproject, the authors chose to restrict this area to 100 m^2^ for a plot due to time limits and poorly studied mycota (much time was spent on collecting and identification of unknown species). The species accumulation curves built by the end of the study showed that all plots were under-studied in terms of species richness (except for the bogged site where the curve approached an asymptote). Moreover, a composite sample made from four coniferous plots (1-4) showed that 400 m^2^ was still not enough to reveal the full species richness (the curve does not reach an asymptote). Thus the observation area of a plot should be significantly increased in the future (1000 m^2^ if possible) for all forest plots. Presently, this drawback was partly fulfilled by random routes which allowed finding a significant portion of species under-revealed through the plot-based study.

The replication of plots is important to study the variation in the fungal communities and to make statistical analyses where 10-20 plots per vegetation type would be a desirable number. However, plot-based studies require large effort and this number is hardly possible unless a group of people are involved. In this case, replicate plots were made in coniferous forests (4 plots) and in secondary aspen forests (3 plots). The analyses showed that replicate plots differed from each other by the number of parameters (species composition, species richness, sporocarps densities etc.). For example, within secondary forests, plot 5 differed markedly by number of species probably due to the differences in micro-habitat conditions. Nevertheless, the differences within a common type was lower than differences between different types of plots as shown by cluster analysis and PCA. In addition, replicate plots showed that differences between replicate plots were higher for fungal communities compared to vegetation (showed by cluster analysis) which shows vegetation being more homogenous compared to fungal communities.

The replicate plots (samples) are also important for construction of species accumulation curves, although this requires a much higher number of plots than used in this study. Instead, micro-plots (5 m^2^) were used as the sample unit to build species accumulation curves and estimate predicted species number.

Frequency and duration are important parameters for a plot-based study design due to the dynamics of sporocarps appearance during the season and through the years. Therefore, a researcher should provide periodical observations of the studied plots (a frequency about 10 days recommended). In this study, one plot or a route per day were visited followed by a day of laboratory work. With this schedule, all 10 plots and 5 routes were visited 5 times per vegetation season (1 time per month from May until September). With regard to the duration of the study, this first year of observation brought preliminary results which will be further continued in the following years of observations. Fortunately, the weather was quite favourable for fungal fruiting during this year with an unusually large amount of precipitation in June-July which allowed a high diversity of species to be revealed.

The expression of quantity of species during a plot-based survey could be done in several ways. Counting of the actual number of sporocarps is laborious but it is the most exact way and it was employed in the study. The resulting data are number of sporocarps per area per visit (sporocarps density) and the sum of visits could create an accumulated sporocarps density during the season. Since the visits were rather rare (part of sporocarps surely appear and vanish in between monthly visits), the accumulated sporocarp density during the season has most likely been under-estimated. A more appropriate characteristic in this case is the maximum species density per observation period. However, nobody has yet estimated an accumulated density of fungal fruiting in the region and this characteristic has been provided as a preliminary estimation.

Sporocarp density is an inappropriate characteristic for further analysis as it counts vegetative structures rather than individuals (genets) and is biased toward species producing many sporocarps per mycelium (e.g. *Pholiota* spp.) vs. species with few sporocarps per mycelium (e.g. *Amanita* spp.). In order to operate with genets, an assumption was used that for most species, sporocarps separated by 10 m or more represent a functional individual for terrestrial macrofungi (Dahlberg and Mueller 2011, O'Dell et al. 2004). Thus, species abundance was counted as micro-plot frequency within a plot. The last quantitative parameter used in the study was species occurrence (number of plots where species was presented), which helped estimate species preferences to certain plots and therefore forest types.

### Species composition

The analysis of taxonomical structure of the total list was undertaken in the authors'previous work Filippova and Bulyonkova 2017. Briefly, the study made a significant impact on the compiled checklist where 284 species were recorded for the first time in vicinities of Khanty-Mansiysk. The total list of terrestrial macrofungi in Khanty-Mansiysk vicinities reached therefore 636 species. About 150 species previously recorded in Khanty-Mansiysk were not registered in present study. Due to varying degrees of study of mycobiota in the nearby regions, only two checklists were chosen to compare the revealed mycobiota: Visimskiy Nature Reserve and Yuganskiy Nature Reserve. The number of agaricoid macrofungi in the Visimskiy Nature Reserve and around Khanty-Mansiysk are more similar (542 vs. 636), while there are fewer recorded species in the Yuganskiy Nature Reserve (325). The abundance of species in major genera is similar in the Visimskiy reserve and around Khanty-Mansiysk, while other genera differ substantially in species abundance. The Jaccard similarity coefficients equal about 0.2 between each three mycobiota. These differences of nearly similar territories pointmost probably to insufficientdegrees of study in each case.

### Phenology

Fruiting phenology in the year of study showed a peak of total sporocarps density in July with a subsequent decline in August and a slight rise in September. The analysis of two major trophic groups separately (ectomycorrhizal and saprotrophic species) showed that the July peak was mostly made up by saprotrophic species (mainly in the fresh cutting site), while ectomycorrhizal species fruited with equal densities in July and August and subsided in September. Fruiting with a single peak at the end of summer is typical for the boreal zone with its short cool summers. However, the observed fruiting pattern could be biased by the unusually high summer precipitation level in 2015 (1.7 and 2.8 times higher than the average in June and July respectively). Subsequent years of plot-based observations may reveal possible correlations between weather parameters and fruiting patterns.

The community composition changed through the season: only 6 species were fruiting in May, 10 species in June, about 50 species in July, the largest set of species fruited during August-September and late fruiting in mid-September was observed in 15 species.

### Analysis of fungal community structure in different forest types

One of the main objectives of the plot-based study was to show the differences between fungal communities in different forest types following clear cut and bogging successions. Although the ten studied plots were located close to one another (within a 5 km radius) and the propagules could easily reach all sites, the fungal communities differed significantly. With regard to clear cut impact, three stages of succession were considered, e.g. 5, 20-30 years after clear cut and intact coniferous forests. The plots located in the forests of different stages were united in three groups by cluster analysis and PCA. The differences between fungal communities of these groups were related to species composition and their relative abundance, but the total number of species in all stages was nearly similar (from 48 to 76 species, 65 species on average). As for the total plot densities of fruiting, the fresh cutting site had nearly twice the number (about 1000 sporocarps/per plot/per season) compared to later stages (equally about 500 sporocarps in aspen forests and in coniferous forests). The fresh cutting site also differed by the highest average number of species within a micro-plot (11 species). The trophic structure of the first stage of succession differed by prevalence of saprotrophic species. A few (6) mycorrhizal species, most probably related to the roots of isolated surviving trees, represented only 3% of total sporocarps density of this plot. Within the saprotrophic group, the fresh cutting site differed by absence of moss saprotrophs (*Galerina* and others) which corresponded to the scarce moss cover observed on the site. In 20-30 years since the clear cut, the density of fruiting halved and the number of species within a micro-plot dropped to an average of 7. Besides, the trophic structure of the species list was made up by about a half of mycorrhizal species and a significant fraction of saprotrophs on moss (despite the still fragmented moss cover in aspen forests). The characteristic species at this stage were saprotrophs of well-developed leaf litter layer (species of *Crepidotus*, *Mycena, Clitocybe, Rhodocollybia*) and two species of *Cortinarius* – symbionts of deciduous trees. The old coniferous forests as the final stage of the succession were characterised by a similar number of species, densities and number of species within a micro-plot as compared to secondary aspen forests. The trophic structure also corresponded to that of aspen forests. Thus, the later stages of succession affected mainly the species composition of the fungal community. The characteristic species at this stage were several mycorrhizal and saprotrophic species.

Ecological succession after logging had been described for ectomycorrhizal (ECM) species by Shubin 1990, Shubin 1998 in the middle taiga zone of the European part of Russia. He describes three main stages of ectomycorrhizal species succession: the first stage includes species such as *Inocybe*, *Laccaria*, *Thelephora* and *Paxillus*; the second stage includes all ectomycorrhizal species except *Cortinarius* and the last stage comprises high diversity of ectomycorrhizal species including diverse *Cortinarius* spp. In these observations, the fresh cutting site was inhabited by 9 species of ECM fungi, mainly associated with isolated surviving trees; secondary aspen forests were inhabited by a total of 51 ECM species, including 11 species of *Inocybe* and 18 species of *Cortinarius*, while old coniferous forests had 82 species of ECM fungi, with a lower number of *Inocybe* (7) and higher number of *Cortinarius* (40 species).

The bogging process is very common in the boreal zone of West Siberia and two plots (9 and 10) were located to study the impact of this factor on the fungal community. The plots were located in proximity and represented different stages of bogging replacing earlier coniferous forest. Although the clustering of vegetation plots showed similarity between these two plots, the fungal community showed another pattern. Plot 10 (wet birch forest) was united with coniferous forest plots while plot 9 (bogged site) significantly differed from the rest of clusters. However, when the separate analysis of trophic groups was applied, plot 10 was united with coniferous forests for saprotrophic species and with a secondary cluster for mycorrhizal species. It is speculated that the the fungal community is more resistant to changes compared to vegetation on the first stages of bogging succession but changes significantly when the bogging is developed. During the initial stages of bogging, the community of ectomycorrhizal fungi changes significantly due the change in the tree canopy. However, the saprotrophic community remains similar to the coniferous forests. The prolonged bogging changes the fungal community drastically. A bogged site differed significantly by number of species (21 vs. 47 in wet forest), lower density (340 vs. 582) and average number of species within a micro-plot (3 vs. 9). The trophic structure differed by a relatively lower number of mycorrhizal species and absence of saprotrophs of humus layer (due to the formed peat layer). When comparing plot 10 with coniferous forests, the total number of species in plot 10 was somewhat lower (47 vs. average 67). The total plot density, number of species within a micro-plot and the trophic structure were nearly identical. The characteristic species of plot 10 were mycorrhizal species of birch (*Russula, Cortinarius*) and several saprotrophic species (*Clitocybe*, *Rickenella*, *Mycena*) probably preferring wet habitats.

### Rare species and conservation

Rarely registered species (singletons, doubletons and species found in random routes) made up more than half of the total species list found during the project, a fact which is partly related to the limited sampling area and short observation time. The rarity of the list was compared with the species occurrence reported in Funga Nordica by Knudsen and Vesterholt (2008). From the authors' list of rare species, 50% were common and very common, 20% were occasional and 16% were rare in boreal zone as reported in Funga Nordica. On the other hand, 9% of the authors' common species were described as rare in FN. The list of these rare species which are also rarely registered within the boreal zone, should be verified from their taxonomical position and then their ecology and protection status should be subjected to special review. Currently, the list amongst others includes three species listed in the Red Data Book of Russia, namely *Polyporus umbellatus*, *Gomphidius flavipes* and *Sarcosoma globosum* (Bardunov and Novikov 2008) and nine species listed in the Red Data Book of Yugra, namely *Arrhenia discorosea*, *Baeospora myriadophylla*, *Clavariadelphus truncatus*, *Cortinarius violaceus*, *Gomphidius flavipes*, *Gomphus clavatus*, *Hericium cirrhatum*, *Hericium coralloides* and *Sarcosoma globosum* (Vasin and Vasina 2013).

### Conclusions

The survey in a poorly explored area in Khanty-Mansiysk vicinities (middle taiga of West Siberia) revealed 460 species of terrestrial macrofungi; 284 of them were recorded for the first time for Khanty-Mansiysk vicinities. The species list differs sufficiently from the lists of adjacent areas indicating insufficient degree of study in each case and the need for further research in our area.

Three hundred and thirteen (313) species were recorded during the plot-based study and an additional 147 species were recorded during random routes near the plots. Although the species accumulation curve built for collective sampling of all plots did not reach an asymptote, the species found during the random survey filled the gap to reach the estimated species number. Despite this theoretical prediction of sufficient degree of diversity surveying, it is believed that many new species of terrestrial macrofungi will be found in the area if surveying continues in the future.

The species diversity of terrestrial macrofungi included about 60% of ectomycorrhizal species forming associations selectively with coniferous (40%) or deciduous trees (30%) or without specialisation to a tree host (30%). The saprotrophic species included plenty of saprotrophs of forest litter (45% of all saprotrophic species) and lignicolous species (26%) while number of saprotrophs of humus was less (18%). These trophic specialisations of macrofungi play a significant role in the carbon and element cycles of the forest ecosystem.

The community of macrofungi showed dynamics in the course of the season: the maximum for fruiting density and the maximum of species number were registered in July with a subsequent decrease in August and slight rise in September. However, the community of ectomycorrhizal species selectively reached their maximum of development for these parameters in July and August and dynamics differed between fungal communities of different vegetation types. Still, the observed fruiting patterns could be biased by the unusually high summer precipitation level during June and July in this year.

The plot-based study revealed differences between communities of terrestrial macrofungi of old coniferous forests, their after-cut secondary formations and bogged stages. During after-cut succession, the number of species does not change significantly, but other parameters undergo significant changes. The community of the fresh cutting site has a higher density and the trophic structure of the community is characterised by prevalence of saprotrophic species. The secondary forest 20-30 years old has the fungal community with reduced density and the number of mycorrhizal species increases. The old coniferous forest is characterised by a similar density and species number as the previous stage, but the composition of ectomycorrhizal species changes to a larger diversity of *Cortinarius* spp. Each stage has its characteristic species of saprotrophic and mycorrhizal macromycetes. The initial bogging changed the community of ecto-mycorrhizal fungi while the saprotrophic community remained similar to the coniferous forest. The developed bogged site has a totally different fungal community: the sporocarp density and number of species dropped and their composition significantly changed.

While estimating the abundance of species in the plot-based study, a large percentage of rare species with 1-2 registrations per survey was found. Species registered during random routes could also be classified as rare and thus the number of rare species reaches 68% of the total list. Part of them (68 species) are rare within all boreal zones based on literature and should be subjected to special attention by conservation programmes. At present, the survey in Khanty-Mansiysk vicinities allowed records to be revealed of 3 species listed in the Red Data Book of Russia and 9 species listed in the Red Data Book of the Yugra region.

## Figures and Tables

**Table 1.** Characteristics of permanent plots of macromycetes observations in different forest types in vicinities of Khanty-Mansiysk, West Siberia.

**Plot No.**	**Coordinate**	**Vegetation type**	**Time after cut, years**	**Trees height and % cover**	**Plants % cover**	**Mosses % cover**	**T, °C for May-September**	**Total number of plant species**	**Dominant plants (only species with >5 % of cover shown)**
**1**	61.08379 N, 69.46695 E	Coniferous mixed forest	-	18-20 m 80%	15	70	Av 11,7 Min 2.3 Max 23	26	*Pinus sibirica, Abies sibirica, Betulapubescens, Picea obovata, Vaccinium myrtillus, Hylocomium splendens, Pleurozium schreberi, Polytrichum commune*
**2**	61.08553 N, 69.47594 E	Coniferous mixed forest	-	20-22 m 70%	25	70		22	*Pinus sibirica, Abies sibirica, Pinus sylvestris, Picea obovata, Vaccinium myrtillus, Linnaea borealis, Hylocomium splendens, Pleurozium schreberi, Polytrichum commune*
**3**	61.08259 N, 69.45434 E	Coniferous mixed forest	-	18-20 m 80%	15	60		26	*Pinus sibirica, Abies sibirica, Picea obovata, Populus tremula, Vaccinium myrtillus, Hylocomium splendens, Pleurozium schreberi*
**4**	61.07960 N, 69.45287 E	Coniferous mixed forest	-	18-20 m 70%	15	75		24	*Pinus sibirica, Picea obovata, Abies sibirica, Populus tremula, Linnaea borealis, Hylocomium splendens, Pleurozium schreberi, Polytrichum commune*
**5**	61.06641 N, 69.46803 E	Aspen forest	25-30	14-16 m 60%	15	3	-	30	*Pinus sibirica, Picea obovata, Abies sibirica, Populus tremula, Gymnocarpium dryopteris*
**6**	61.06636 N, 69.47094 E	Aspen forest	25-30	16-18 m 70%	25	5		31	*Populus tremula, Betula pubescens, Pinus sibirica, Vaccinium vitis-idaea, Equisetum sylvaticum*
**7**	61.05473 N, 69.42897 E	Aspen forest	20-25	14-15 m 70%	15	10	Av 12.2 Min 1.9 Max 30	33	*Populus tremula, Betula pubescens, Polytrichum commune, Pleurozium schreberi*
**8**	61.05523 N, 69.41694 E	Fresh cutting site	5	1-3 m 30%	40	5	Av 12,4 Min -2,4 Max 32,9	37	*Populus tremula, Gymnocarpium dryopteris, Vaccinium vitis-idaea, Equisetum sylvaticum, Calamagrostis canescens*
**9**	61.05791 N, 69.43936 E	Bogged site	-	3-4 m 30%	40	90	Av 13,4 Min -0,6 Max 36,8	22	*Betula pubescens, Carex globularis, Carex lasiocarpa, Calamagrostis purpurea, Sphagnum angustifolium, Polytrichum strictum*
**10**	61.05746 N, 69.44044 E	Birch forest	-	8-10 m 80%	8	80	Av 11,9 Min -0,4 Max 26,4	27	*Betula pubescens, Vaccinium vitis-idaea, Polytrichum commune, Pleurozium schreberi, Polytrichum juniperinum*

**Table 2.** Sporocarps densities by plots counted once in each month, number of sporocarps/100 m^2^

**Plots**	**May**	**June**	**July**	**August**	**September**	**All season, sum**
Plot 1	1	4	153	136	201	495
Plot 2		2	165	126	222	515
Plot 3	41	22	98	123	193	477
Plot 4	24	18	192	139	125	498
Plot 5	3	5	75	115	109	307
Plot 6	26	8	66	188	228	516
Plot 7	6	23	256	90	194	569
Plot 8	25	40	728	79	138	1010
Plot 9	32	26	202	21	59	340
Plot 10	5	6	348	190	33	582
Sum, sporocarps/1000 m^2^	163	154	2283	1207	1502	5309

## References

[B3767595] Arnolds E. (1992). The analysis and classification of fungal communities with special reference to macrofungi. fungi in Vegetation Science, Handbook of Vegetation Science.

[B3767794] Bardunov L, Novikov V (2008). Krasnaya kniga Rossiyskoy Federatsii (rasteniya i griby) (Red book of Russian Federation (Plants and fungi)).

[B3767605] Borcard D., Gillet F., Legendre P. (2011). Numerical ecology with R.

[B3767614] Bulatov V. (2007). Geografiya i ekologiya goroda Khanty-Mansiyska i ego prirodnogo okruzheniya (Geography and ecology of Khanty-Mansiysk and its surroundings).

[B3767646] Burova L (1986). Ekologiya gribov makromitsetov (Ecology of macromycetes).

[B3767655] Colwell R. K., Chao A., Gotelli N. J., Lin S. Y., Mao C. X., Chazdon R. L., Longino J. T. (2012). Models and estimators linking individual-based and sample-based rarefaction, extrapolation, and comparison of assemblages. Journal of Plant Ecology.

[B3767668] Dahlberg A., Mueller G. M. (2011). Applying IUCN red-listing criteria for assessing and reporting on the conservation status of fungal species. Fungal Ecology.

[B3767678] Filippova N. (2017). The communities of terrestrial macrofungi in different forest types of boreal zone in West Siberia. Version 1.1. Yugra State University Biological Collection (YSU BC) accessed via GBIF.org on 2017-08-14. Sampling_event Dataset.

[B3767698] Filippova N. V., Bulyonkova T. M. (2017). The diversity of larger fungi in the vicinities of Khanty-Mansiysk (middle taiga of West Siberia). Environmental Dynamics and Global Climate Change.

[B3767708] Il'ina I. S., Lapshina E. I., Lavrenko N. N., Mel'tser L. I., Romanova E. A., Bogoyavlenskiy B. A., Makhno V. D. (1985). Rastitel'nyy pokrov Zapadno-Sibirskoy ravniny (Vegetation of West Siberian Plain).

[B3767720] Kalamees K. (1968). Mycocoenological methods based on investigations in the Estonian forests. Acta Mycologica.

[B3932396] Kirk P. M., Cannon P. F., Minter D. W. (2008). 10th edition. Ainsworth & Bisby's Dictionary of the fungi.

[B3767730] Knudsen H., Vesterholt J. (2008). Funga Nordica: agaricoid, boletoid and cyphelloid genera.

[B3767739] Kovalenko A. E. (1980). Ecological review of fungi order Polyporales s.str., Boletales, Agaricales s.str., Russulales in the mountain forests of the central part of the North-West Caucasus. Mycology and Phytopathology.

[B3767749] Lodge D. J., Ammirati J. F., O’Dell T. E., Mueller G. M., Huhndorf S. M., Wang C. J., Stokland J. N., Schmit J. P., Ryvarden L., Leacock P. R., Mata M. I., Mueller G. M. (2004). Terrestrial and lignicolous macrofungi. Biodiversity of fungi. Inventory and monitoring methods.

[B3767770] O'Dell T. E., Lodge J. D., Mueller G. M., Mueller G. M. (2004). Approaches to sampling macrofungi. Biodiversity of fungi. Inventory and monitoring methods.

[B3932208] Oksanen J., Blanchet F. G., Kindt R, Legendre P., Minchin P. R., O'Hara R. B., Simpson G. L., Solymos P., Stevens M. H. H., Wagner H. (2013). Vegan: Community Ecology Package. R package version 2.0 - 7. Available from.

[B3932197] Team R Core (2015). R: A language and environment for statistical computing. R Foundation for Statistical Computing.

[B3932234] worldwide R Core Team and contributors (2017). Team and contributors worldwide. The R Stats Package, version 3.5.0.

[B3767803] Shubin V (1990). Makromitsety lesnykh fitotsenozov taezhnoy zony i ikh ispol'zovanie (Forest macromycetes of taiga zone and their usage).

[B3767812] Shubin V (1998). Ecological niches and succession of symbiotrophic macromycetes in forest ecosystems of taiga zone. I. Ecological niches. Mycology and Phytopathology.

[B3767822] Vasin A, Vasina A (2013). Krasnaya kniga Khanty-Mansiyskogo avtonomnogo okruga - Yugry (Red book of Khanty-Mansiykiy Autonomous Okrug - Yugra).

